# Geographical weighted regression analysis of delayed antenatal care initiation and its factors among all reproductive-aged women in Ethiopia, 2016

**DOI:** 10.1186/s40834-022-00190-z

**Published:** 2022-11-01

**Authors:** Abiyu Abadi Tareke, Kassahun Dessie Gashu, Berhanu Fikadie Endehabtu

**Affiliations:** 1 Department of monitoring and evaluation, west Gondar zonal health department, Gondar, Ethiopia; 2 University of Gondar College of medicine and health sciences department of health informatics, Gondar, Ethiopia

**Keywords:** Delayed antenatal care, Geographically weighted regression, EDHS, Ethiopia, 2016

## Abstract

**Background:**

Delayed antenatal care is when the first visit is carried out after 12 gestational weeks. Despite the fact that many studies have been conducted on antenatal care initiation, little attention has been paid to its spatial pattern. Therefore, this study examine geographical weighted regression analysis of delayed antenatal care initiation and its factors among all reproductive-aged women in Ethiopia.

**Objective:**

To assess geographical weighted regression analysis of delayed antenatal care initiation and its factors among all reproductive-aged women in Ethiopia, 2016.

**Methods:**

This study was grounded on the 2016 Ethiopian Demographic Health Survey. It incorporated extracted sample size of 4740 (weighted) reproductive-aged women. ArcGIS version 10.8 and SaTScan™ version 9.7 software were employed to investigate geographic information. To distinguish factors associated with hotspot areas, local and global models were fitted.

**Result:**

the geographic pattern of Delayed antenatal care initiation was clustered (Moran’s I = 0.38, p < 0.001). Kuldorff’s spatial scan statistics discovered three significant clusters. The most likely cluster (LLR = 66.13, p < 0.001) was situated at the zones of SNNP and Oromia regions. In the local model, being uneducated, being poor wealth, having an unwanted pregnancy, and having higher birth order were factors associated with spatial variation of delayed antenatal care.

**Conclusion:**

The spatial pattern of delayed antenatal care in Ethiopia is clustered. Maternal education, wealth status, pregnancy desirability, and birth order were predictor variables of spatial variation of delayed antenatal care. Therefore, designing a hotspot area-based interventional plan could help to improve early ANC initiation.

## Introduction

The World Health Organization (WHO) defines maternal health as the health of women starting from preconceptions extending to pregnancy, time of birth, and the postpartum period [1]. Antenatal care (ANC) is scheduled, client-centered obstetric care focused on the wellbeing of maternal-fetal through regular monitoring of pregnancy. It is the first and foremost element of maternal health care, which is tailored to promote health, prevent diseases, provide conducive birth preparedness situations, and treat preexisting maternal health problems which worsen during the time of pregnancy [2].

Early provision of first antenatal care services has a substantial contribution in minimizing maternal mortality rate through early identification and treatment of potential risks [3]. The focused antenatal care model listed in the WHO clinical guideline recommends that the first antenatal care need to be initiated before 12 weeks of gestation [2]. This gives the opportunity to the service providers enough time to detect and treat prior health conditions like hypertension, anemia, malaria, syphilis, and HIV/AIDS [4]. Even if Ethiopia showed good progress in the decrement of maternal deaths by 71.8% from 1990 to 2015, the burden is still at high levels [5] i.e. 412 deaths for every 100,000 live births [6]. This indicates more efforts are needed to halt this high rate. Early provision of antenatal care is one of the strategies to bring down maternal and neonatal death [7].

Delayed ANC is among the driving forces to devastative pregnancy outcomes such as placental abruption, intrauterine infections, preterm birth, low birth weight, intrauterine fetal death, maternal and neonatal death [8, 9]. Even though antenatal care is exempted services, evidence from low-income countries demonstrated that too many women failed to attend their antenatal care initiation on time [10–13]. In Ethiopia, the burden of delayed antenatal care initiation is tremendous, ranging from 53 to 86% [11, 14–16]. Respective studies have examined various factors that influence delayed ANC initiation such as: age [15, 17, 18], place of residency [17], maternal education [18], paternal education [19, 20], marital status [12, 17, 21, 22], maternal occupation [12, 19, 20, 23], wealth status [15, 18], family size, perception of distance to health facility [19, 20, 22, 24] and enrollment to health insurance [22, 25–27], women’s autonomy to visit health facility [28], place of residency [13, 25], media exposure [25, 29, 30], birth order [22, 31], gravidity [32] and having unplanned pregnancy [20].

In Ethiopia, many studies have adopted global regression models to deal with delayed ANC. However, the global models might not have addressed the spatially varying relationship between multiple predictors and the occurrence of delayed ANC initiation because of the constant coefficients in the overall study area. Evidence about the spatial pattern of delayed ANC in Ethiopia is limited. Therefore, this study aimed at exploring the spatial pattern of delayed ANC initiation. Moreover, spatial analyses are very important in identifying geographic locations which have a higher magnitude of delayed ANC (hotspot areas) and facilitate location-based interventions.

## Methods and materials

### Study design and sampling procedures

This study utilized data from the 2016 EDHS and which was carried out in Ethiopia. Ethiopia is located in the North-Eastern part of Africa. Which is situated at between 3^0^ and 15^0^ north latitude, and 33^0^ to 48^0^ East latitude, with a total area of 1.13 × 106 km2. The nation is divided into nine administrative regions (Tigray, Afar, Amhara, Oromia, Somalia, Benishangul, Southern Nations & Nationality, and People’s (SNNP), Gambela, Harari) and two City Administration councils of Dire Dawa and Addis Ababa for administration purposes. The regional states and city administrations are further divided into zones, woredas (districts), and Kebeles (sub-districts which is the bottommost administrative hierarchy in the country) [33].

In 2016 EDHS, two-stage cluster sampling was applied. In the first stage, 645 clusters (202 in urban and 443 in rural area) were randomly selected proportional to their EA size, and in the second stage, 28 households were selected from each selected EAs. A weighted sample of 4,740 reproductive-aged women [33]. The dataset type utilized to analyze this manuscript was kids’ record (KR). The KR dataset was utilized because this dataset contains data of the most recent of interviewed mothers/caregivers, born in the five years preceding the survey. Only the most recent birth is included in this study to avoid outdated birth related demographic information and this helps minimize recall bias.

### Study variables

#### Dependent variable

The dependent variable for this study was Delayed ANC initiation (delayed or not).

##### Delayed antenatal care initiation

women are said to be “delayed” If they attend their first antenatal care visit after 12 gestational weeks, otherwise they are said to be “early”[2, 34].

#### Independent variables

After an extensive literature review, maternal age, maternal education, marital status, occupation, partner education, wealth status, perceived distance, media exposure, family size, health insurance ownership, abortion history, and autonomy in decision making were considered independent variables for this particular study.

##### Media exposure

Created by combining whether a respondent reads the newspaper, listens to the radio, and watches television. If the study subject was exposed to at least one of the three media labeled as “exposed” and coded as” 1”, otherwise “not exposed” coded “0”.

### Data management

The data was changed suitably a way through editing, verifying, arranging, and recoding by using STATA/SE version 16.0. The proportion of each dependent and independent variables was extracted by cross tabulating with the variable cluster number (v001) and saved to Excel as a CSV file. Variables that had a linear relationship with the outcome variable were imported to Arc GIS 10.8 to fit the ordinary least squares (OLS). To account for the effect of the complex sampling design of the survey/hierarchical nature of EDHS dataset, to restore the representativeness of the survey and to get reliable statistical estimates, the data were weighted using the “svyset” STATA command. This command was applied as a prefix to every analysis of this study.

### Statistical analyses

#### Spatial autocorrelation

Arc GIS version 10.8 software was used to investigate the presence of spatial variation and map model parameters between local models. The global spatial autocorrelation (Global Moran’s I) was calculated to declare whether delayed ANC initiation was dispersed, clustered, or randomly distributed in Ethiopia [35, 36]. Global Moran’s I is a spatial statistic used to measure spatial autocorrelation by taking the entire dataset and producing a single output value that ranges from − 1 to + 1. Moran’s output closer to − 1 indicates that the event of interest is dispersed, whereas closer to + 1 indicates clustering, and if closer to 0 it implicates a random pattern. A statistically significant Moran’s I (p < 0.05) shows that the distribution of delayed ANC initiation is nonrandom (either clustered or dispersed) [37]. Additionally, semivariogram was modeled to verify and quantify the spatial clustering of delayed ANC initiation in Ethiopia. Furthermore, semivariogram was utilized to develop model fitness parameters, which helped us in selecting the best interpolation model for this dataset.

#### Incremental spatial autocorrelation

maximum peak distance from the spatial incremental autocorrelation model indicates that at which the spatial dependence of delayed ANC was most prominently significant and we used this maximum peak distance value as threshold distance for hotspot analysis [38].

#### Spatial interpolation

The ordinary Kriging method of spatial interpolation was applied to predict the proportion of delayed antenatal care initiation of un-sampled locations based on neighborhood measured values. Kriging method was used in favor of other interpolation techniques for the reason of Kriging interpolation is an optimal interpolator offering a minimum mean error (ME) and root mean square error (RMSE) [39].

#### Spatial scan statistical analysis

Bernoulli and purely spatial Kulldorff’s scan statistics analysis was utilized. Only areas with a high risk of prevalence were applied to determine the geographical locations of statistically significant clusters of delayed ANC initiation using SaTScan™ version 9.7 software. We used the Bernoulli model because the data is binary (delayed or not). Delayed ANC initiation was taken as case (1) and not delayed was considered as non-case (0). The case file (1), non-case file (0), and coordinate (latitude and longitude) file were imported to SaTScan™ software to find the location of the significant clusters. The maximum scanning window size was scaled in terms of percentage of the total population at risk. To avoid missing out very small and very large-sized clusters, the maximum geographic cluster size was adjusted to < 50% of the population at risk as an upper limit. Most likely (primary), clusters were identified using p-value and likelihood ratio tests. The cluster with the maximum likelihood ratio constitutes the most likely cluster [40].

### Factors associated with delayed ANC initiation

#### Ordinary least squares (OLS) model

OLS is a global regression model that uses a single equation to estimate the relationship between the dependent and independent variables, and it assumes the coefficients of each variable are homogenous across the study area [41]. The OLS model is the first step toward choose the appropriate predictor variables for the spatial variation of delayed ANC initiation [42]. Before fitting the global and local regression model, confirmation of no stationary proportion of delayed ANC initiation is obligatory. The spatial no stationary was ascertained through the use of global spatial autocorrelation. Subsequently, global spatial regression modeling was calibrated to identify factors associated with the proportion of delayed ANC initiation.

The six assumptions of the OLS model (the explanatory variables should have the relationship, we expected, the significance of each explanatory variable, the randomness of residuals, assuring the statistical no significance of Jarque-Bera statistics, VIF value, and the strength of R-square) were checked before proceeding to the local model. The multicollinearity was assessed based on the values of the Variance Inflation Factor (VIF). Predictors with VIF values greater than 7.5, i.e., cut point to declare the presence of multicollinearity and no sign of multicollinearity was observed in this data.

After checking the assumption of the OLS model, the local model, i.e., GWR is a local form of OLS used to model spatially varying relationships, which assumes that the relationship between variables varies spatially [43] and it was implemented through the use of the new version of GWR called Multiscale Geographically Weighted Regression (MGWR) version 2.2.1 software. Classic GWR assumes that the processes being modeled occur on the same spatial scale. However, MGWR allows different processes to operate at different scales [44]. Unlike the classic GWR, MGWR avoids a single bandwidth assumption for all covariates and allows covariate-specific bandwidth [45]. The spatial kernel was adjusted with adaptive bisquare in preference to the fixed Gaussian kernel because of its minimum yield of AICc value. Additionally, AICc and Adjusted R2 were utilized as model selection criteria between the global regression (OLS) and local regression (MGWR). The model with the lowest AICc and higher Adjusted R2 was preferred as best fitted [42].

### Ethical consideration

Through using the DHS measure website http://www.dhsprogram.com/ to access Ethiopian 2016 EDHS Dataset and Global Positioning system (GPS) data, registration was conducted and an authorization letter was gotten to access those requested tools. Accordingly, all necessary data were downloaded from the Demographic and Health Surveys (DHS) Program web page. However, as the authors’ utilized secondary dataset from 2016 EDHS participant consent is not required.

## Results

### Characteristics of the study participants

A total of 4740 women who gave birth within five years preceding the survey and had ANC visits were included. The overall prevalence of delayed ANC in Ethiopia was 67.3% (95%CI: 66–68.6%). The median gestational age at which the first ANC visit was conducted was 4 months (IQR = 3–5). The majority of the rural residents (71%) and not educated women (70%) had delayed ANC initiation. More than two-thirds of married women experienced delayed ANC initiation (Table 1).

**Table 1:** descriptive characteristics of the study participants (n = 4740) in Ethiopia, 2016

Variables	Delayed ANC initiation	
	**Yes**	**No**
**Maternal Age**		
15–24	822(67.0%)	403(33.0%)
25–34	1658(67.0%)	830(33.0%)
35–49	710(69.0%)	317(31.0%)
**Place Of Residency**		
Rural	2743(71.0%)	1123(29.0%)
Urban	447(51.0%)	427(49.0%)
**Religion**		
Orthodox	1218(60.5%)	795(39.5%)
Muslim	1048(67.0%)	516(33.0%)
Protestant	836(80.0%)	207(20.0%)
Others	88(74.0%)	31(26.0%)
**Educational Status**		
No Education	1818(70.0%)	737(29.0%)
Primary	1042(66.3%)	529(33.7%)
Secondary	219(56.5%)	168(43.5%)
Higher	111(49.0%)	116(51.0%)
**Wealth status**		
Poorest	552(70.0%)	235 (30.0%)
Poorer	676(72.6%)	255(27.4%)
Middle	688(70.0%)	295(30.0%)
Richer	683(70.0%)	280(29.0%)
Richest	591(55.0%)	484(45.0%)
**Marital Status**		
Married	2986(68.0%)	1412(32.0%)
Not Married	204(59.6%)	138(40.4%)
**Media Exposure**		
Not Exposed	1924(70.7%)	798(29.3%)
Exposed	1266(62.7%)	752(37.3%)
**Birth order**		
Primi-para(one)	680(61.2%)	431(38.8%)
Multipara(2–4)	1355(65.0%)	729(35.0%)
Grand Multipara(5+)	1156(74.8%)	389(25.2%)
**Latest Pregnancy Desirability**		
Wanted	2410(67.0%)	1194(33.0%)
Mistimed	545(66.4%)	275(33.6%)
Unwanted	236(74.5%)	80(25.5%)
**Ever Had Of a Terminated Pregnancy**		
Yes	301(69.3%)	134(30.8%)
No	2889(67.0%)	1416(33.0%)
**Perceived distance to health facility**		
Big Problem	1684(70.7%)	698(29.3%)
Not Big Problem	1506(64.0%)	852(36.0%)
**Health insurance enrollment**		
Yes	133(55.80%)	105(44.2%)
No	1445(32.1%)	3058(67.9%)

Others*: catholic, traditional, others

**Fig. 1:**
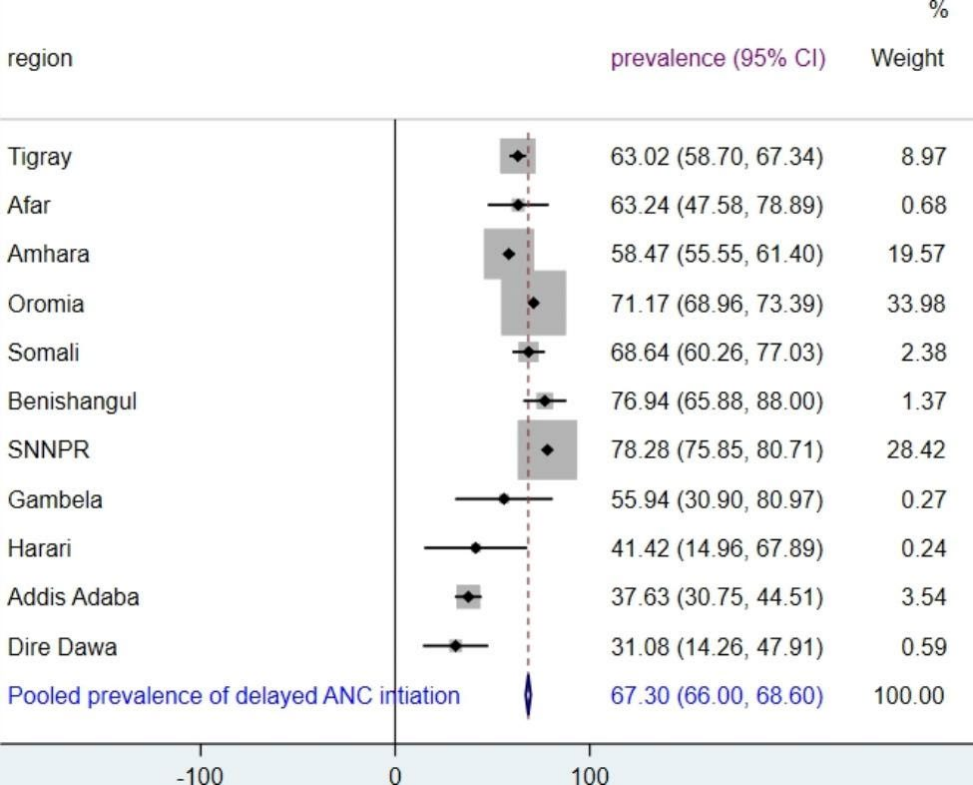
forest plot of prevalence of delayed ANC in Ethiopia, 2016

There were regional variations in the prevalence of delayed ANC initiation; higher in SNNP (78%), Benishangul (77%), Oromia (71%), and Somalia (68.6%), and the prevalence was lower in Dire Dawa (31%), Harari (41.4%) and Addis Ababa (37.6%). To sum up, the prevalence of delayed ANC initiation was found higher than the national pooled prevalence (point estimate right of the red line) in Oromia, Somalia, Benishangul, and SNNNP regions (Fig. 1).

### Spatial distribution of delayed ANC initiation

From the incremental spatial autocorrelation model, a total of 12 distance bands were detected by calibrating the beginning distance to 140,000 m, which generated 265,663 m of the first peak and 391,327 m of a maximum peak, at which the clustering of delayed ANC initiation was more manifested. Hotspot analysis was conducted by using the maximum peak distance as the threshold distance band.

The assumption that things nearby tend to be more similar than things that are farther apart was explored and quantified through the use of the semivariogram model. The semivariogram depicts the spatial autocorrelation of the measured sample points. The lower the Variogram value, the more interdependence of observations (autocorrelation).

**Fig. 2:**
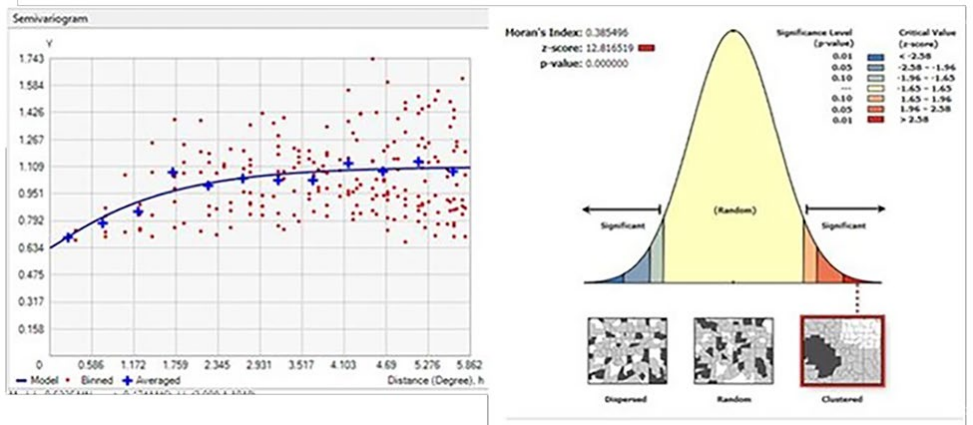
optimized Variogram (left) and global spatial autocorrelation (right) result of delayed ANC initiation in Ethiopia, 2016

In Fig. 2, the left side graph depicts that as the distance increases (x-axis), the value of Variogram (y-axis) increases, indicating the tendency to have spatial clustering of delayed ANC initiation. Value of characteristics of the Variogram model, i.e., nugget (the distance where the model first flattens out), sill (Variogram value at which autocorrelation stops) and range (distance at which spatial autocorrelation stops) were 0.5931271, 1.02033, and 5.53 respectively. The spatial dependence “nugget-to-sill ratio” for delayed ANC initiation was 58% (lay between 25% and 75%), which corresponds to a moderate degree of spatial dependence [46]. Parameters of the semivariogram model were used for ordinary Kriging interpolation model development. The result of the semivariogram was also supported by the spatial autocorrelation model, which found that the spatial distribution of delayed ANC visits was nonrandom (global Moran’s value = 0.385, p-value < 0.001). This confidence level indicates that the probability of randomness of delayed antenatal care initiation is less than 1%, and the positive signed Moran’s index value symbolizes the clustering of delayed ANC initiation in Ethiopia. (Fig. 2).

### Spatial interpolation of delayed ANC initiation

A comparison of inverse distance weighting (IDW) and ordinary Kriging (OK) performance was done using mean error (MPE) and root mean square error (RMSPE). The ME value was − 0.01583 for IDW and 0.002568 for OK, indicating a satisfactory MPE for both models (values were close to zero). Based on the exponential semivariogram model, RMSPE of the OK model was lower, i.e., 0.2245, than RMSE of IDW, i.e., 0.2357, suggesting that the ordinary Kriging accurate model to interpolate the delayed ANC (Table 2).

**Table 2:** selection of the best interpolation model for predicting delayed ANC in Ethiopia, 2016 based on the exponential semivariogram model

Interpolation method	Mean predicted error (MPE)	Root mean squared predicted error (RMSPE)
**Deterministic methods**		
Inverse distance weighting (IDW)	-0.01583	0.235704
**Geostatistical methods**		
Ordinary Kriging	0.002568	**0.2245674**
Simple Kriging	-0.006485	0.2260122
Universal Kriging	0.002568	0.2248674
Indicator Kriging	-0.0008834	0.4426523
Probability Kriging	-0.00027244	0.43963
Disjunctive Kriging	-0.0005645	0.226032

**Fig. 3:**
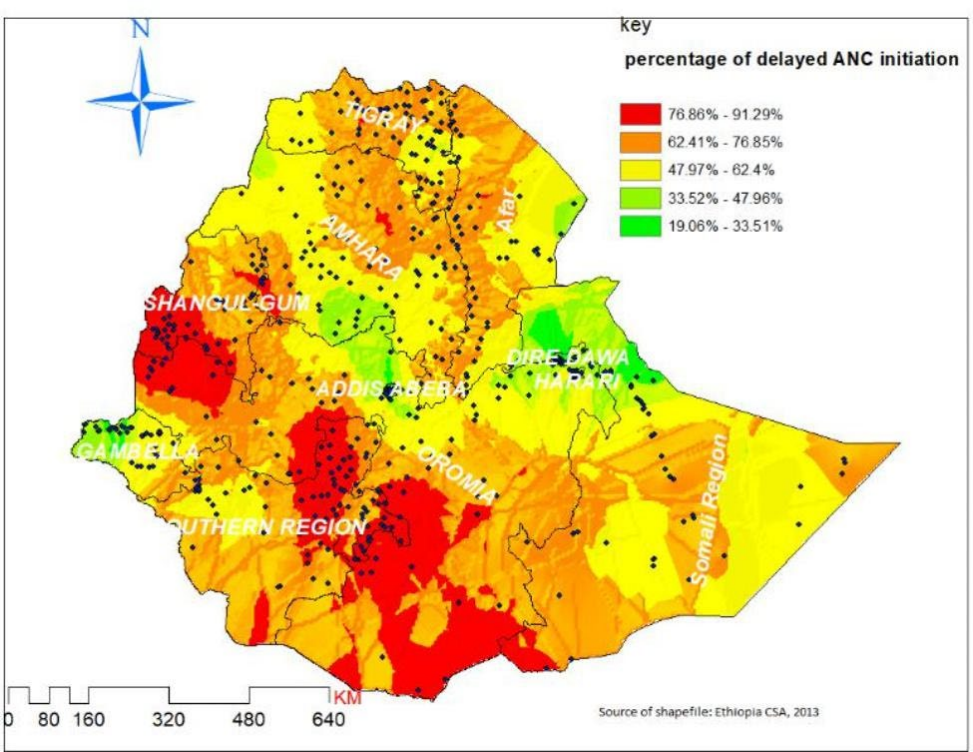
Ordinary Kriging of interpolation of proportion of delayed ANC initiation among reproductive age women in Ethiopia, 2016 EDHS.

Using Kriging interpolation, a higher proportion of predicted delayed ANC initiation (red shaded) was found in Central, Southern, and Western parts of Oromia, Southern, East and Northern part of SNNP, Eastern and southwest of Benshangul-gumuz, the central and northern part of Tigray, northwest, and southwest of Afar, and central, west and east parts of Somali regions. Conversely, the predicted low delayed ANC initiation (green shaded) covers most parts of Northern Somalia, Western segment Gambela, and Southern Amhara and Northern Oromia regions (Fig. 3).

### Hotspot and cold spot analysis of delayed ANC initiation

**Fig. 4:**
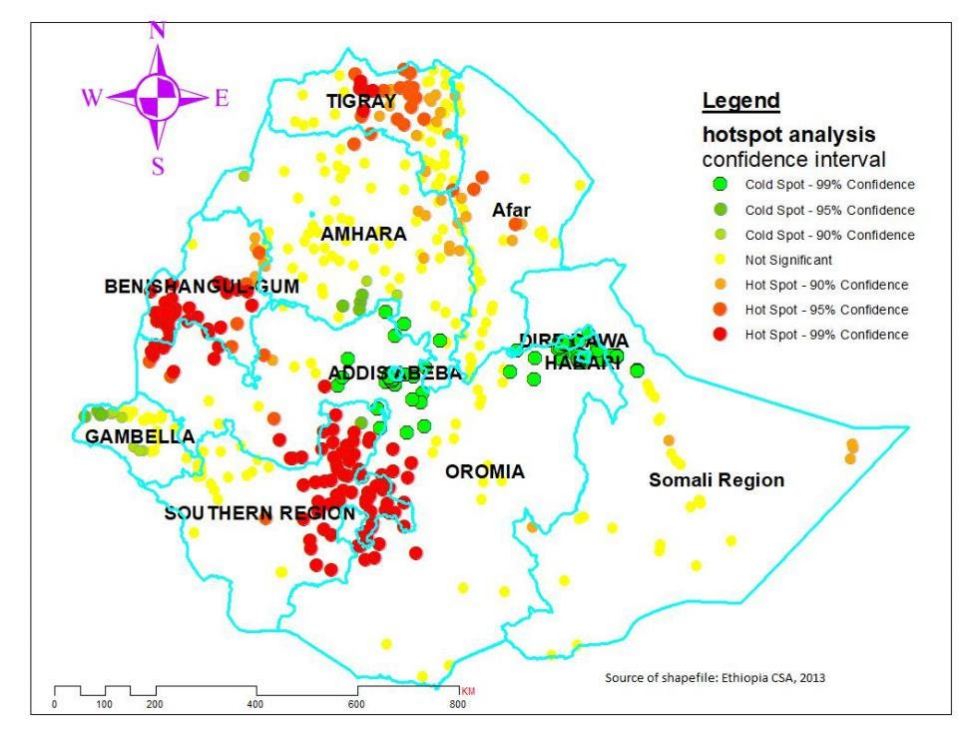
hotspot and cold spot analysis of proportion of delayed antenatal care initiation in Ethiopia, 2016

The higher proportion of delayed ANC initiation (red dots), which concentrated at the central, northwest, and eastern part of Tigray, North East of SNNP, most parts of Benishangul Gumuz and some parts of West Afar, East part of Somalia, southwestern Oromia, and northeast of Amhara region. Conversely, a Lower proportion (green dots) was detected in central and eastern Oromia, southern & western parts of Amhara, Harari, Dire Dawa, and the western part of the Gambela region (Fig. 4).

### Spatial scan statistics of delayed ANC initiation

Kulldorff’s spatial scan statistics identified three statistically significant clusters of delayed ANC initiation using 50% of the total population as the maximum spatial circular window size. The most likely cluster (LLR = 66.13, p-value < 0.001) with a total enumeration area of 92 was found concentrated in the SNNP region mainly in the whole part of (Gurage zone, Hadiya zone, Simen Omo zone, Amaro zone, Gedeo zone, Kembata Alabat zone, Sidama zone (currently Sidama region), yem zone, Burji zone, and Konso zone) and southeast portion of Keficho Shekich zone, the eastern part of Benchi Maji zone and northern part of Debub Omo zone. This primary cluster also covers the Oromia region (i.e., the whole portion of Jimma Zone, the eastern part of Illu-Aba-Borra Zone, the southern part of East Wollega, the southern portion of West and East Shoa Zone, southwest of Arsi Zone, some areas of the west part of Bale zone, and northwest of Borrena zone). The primary cluster was centered at a longitude of 7.132513 N, latitude 37.536830 E, 214.40 km radius, and a relative risk of 1.37 (Table 3). This notices that women within the spatial window had a 37% elevated risk of delayed ANC initiation than women outside the window.

**Fig. 5:**
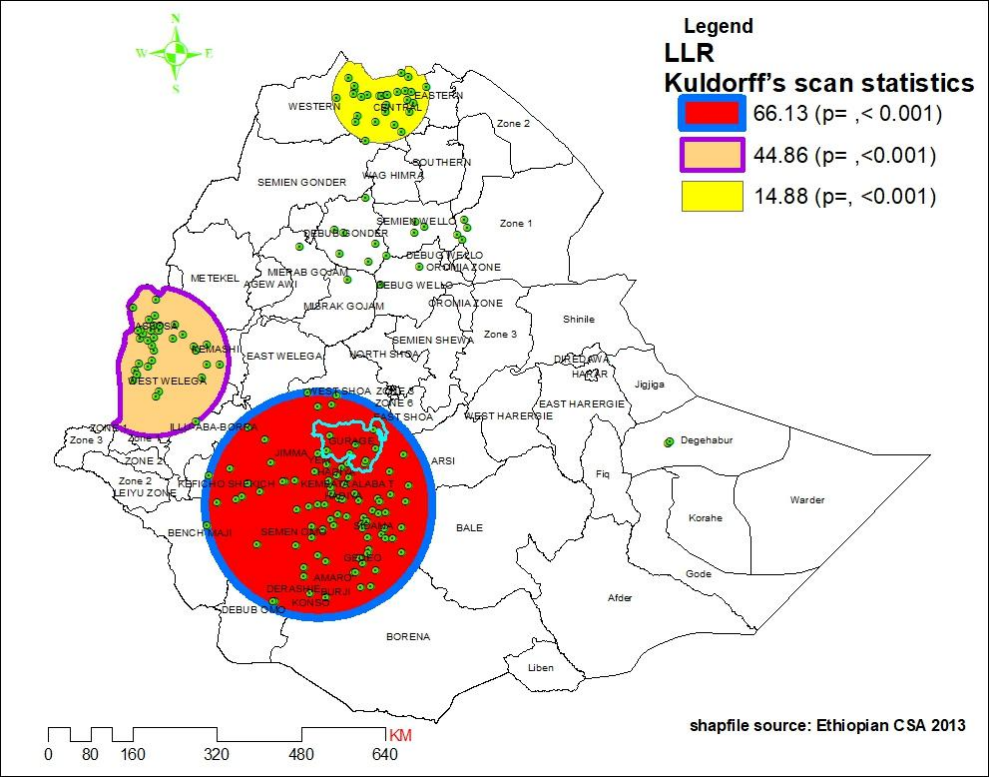
most likely and secondary clusters with high rate of delayed antenatal care initiation in Ethiopia, 2016

In addition to the most likely cluster, two statistically significant secondary clusters were discovered in the northern and western parts of Ethiopia. The secondary cluster 1 was situated in western Ethiopia (LLR = 44.86, p-value < 0.001, and RR = 1.43), which incorporated a total of 35 enumeration areas. It predominantly covers the western Wollega zone, the western portion of Kemashi zone, and the whole portion of Assossa zone and is centered at 9.59 North latitude and 34.70 east longitude and is 141.53 km radius. The second secondary cluster (i.e., secondary cluster 2) scanning window was located in the central and western zone of the Tigray region. It was centered at 14.123765 N, 38.589911 E with 89.50 km radius, and LLR of 15 at p-value < 0.001 (Fig. 5). It showed that pregnant women within the spatial window had 1.25 times high risk of delayed ANC initiation than women outside the window (Table 3).

**Table 3:** Cluster detection analysis results for delayed ANC initiation in Ethiopia, 2016, using the Bernoulli model

Cluster	N	Latitude	Longitude	Radius (KM)	Pop.	Case	RR	LLR	p-value
Most likely	92	37.536830	7.132513	214.4	810	634	1.37	66.13	< 0.001
Secondary 1	32	34.700066	9.590824	141.53	313	265	1.43	44.86	< 0.001
Secondary 2	28	38.589911	14.123765	89.5	313	235	1.25	14.88	0.001

Note: RR = relative risk, LLR = log-likelihood ratio, N = number of clusters (EA), pop. =population, N = number of enumeration areas incorporated inside the scan window

### Spatial regression of the predictors of delayed ANC initiation

#### Ordinary least squares (OLS) model results

The ordinary least squares (OLS) model was fitted to identify factors associated with spatial variation and delayed ANC initiation. Being uneducated, being from a poor wealth status, household, having unplanned last pregnancy, and having higher birth order were found associated with delayed ANC. No signal of multicollinearity was noticed among the identified independent variables (mean VIF = 1.4025, minimum VIF = 1.03, and maximum VIF = 1.59) (Table 4).

The adjusted R2 = 0.22.7 from OLS global model output indicated that 22.7% of the variation in delayed ANC initiation was explained by the four explanatory variables (Table 4). The significant joint F-statistic and joint Wald statistic indicated that there is a significant linear relationship between the dependent variable and the independent variables. The p-value > 0.05 of Jarque-Bera statistics indicates that the model prediction using OLS was free from biased (the assumption of normality of residuals was fulfilled). The Koenker statistics in the model had a statistically significant p-value, indicating that the regression model is inconsistent across the study area (with the change in geographic position the relationship of variables will also change). This suggests that the GWR model was considered more appropriate to estimate the model parameters.

**Table 4:** The Ordinary Least Squares (OLS) regression result summary

Variables	Coefficients	Robust t-statistics	Robust probability	VIF
Intercept	0.247	6.47	0.000000*	-----
Not educated women	0.270	5.30	0.000000*	1.59
Women from poor wealth status	0.182	3.82	0.000154*	1.44
unplanned last pregnancy	0.104	2.82	0.004900*	1.03
Birth order 4 and above	0.140	2.80	0.005185*	1.55
**Ordinary least square regression Diagnostics**				
Number of data points	606	--p-value		
Adjusted R Squared	22.7%	----		
Joint F-statistics	45.47	0.000000*		
Joint Wald statistics	158.97	0.000000*		
Koenker (BP) statistics	13.79	0.007995*		
Jarque – Bera	4.68	0.096447		

*: Significant at the 0.05 level

### Geographically weighted regression analysis

Comparing the two models using diagnostic parameters (AICc and R^2^), AICc was reduced from 1569.5 (for OLS model) to 1398.4 (for MGWR model). The Adjusted R^2^ increased from 0.227(22.7%) in the OLS model to 0.46 (46%) in the MGWR model. Therefore, the diagnostic parameters of MGWR are favorable, indicating that Multiscale geographic weighted regression (local model) is superior to OLS (global model) (Table 5).

**Table 5:** Model comparison of OLS and GWR model fit/performance of delayed ANC initiation in Ethiopia, 2016

fitness parameter	OLS model	MGWR Model
AICc	1569.5	1398.4
R-squared	0.232(23.2%)	0.499(50%)
Adjusted R-squared	0.227(22.7%)	0.46(46%)

Table 6 depicted that the mean and median beta coefficient of all explanatory variables of spatial variation of delayed ANC initiation were positive. Indicating a positive association between delayed ANC initiation and the four explanatory variables (Being not educated, poor wealth status, unplanned last pregnancy & having higher birth order).

**Table 6:** Summary of GWR coefficients describing the spatially varying relationships between delayed ANC initiation and socio-economic obstetric factors in Ethiopia, 2016

Variables	Mean	SD	Min	Median	max
Intercept	0.023	0.421	-1.008	0.065	0.811
Being not educated	0.147	0.115	-0.032	0.169	0.366
poor wealth status	0.174	0.172	-0.147	0.234	0.395
unplanned last pregnancy	0.063	0.007	0.046	0.062	0.076
birth order 4 and above	0.105	0.076	-0.016	0.073	0.24

SD = standard deviation, min = minimum, and max = Maximum

**Fig. 6:**
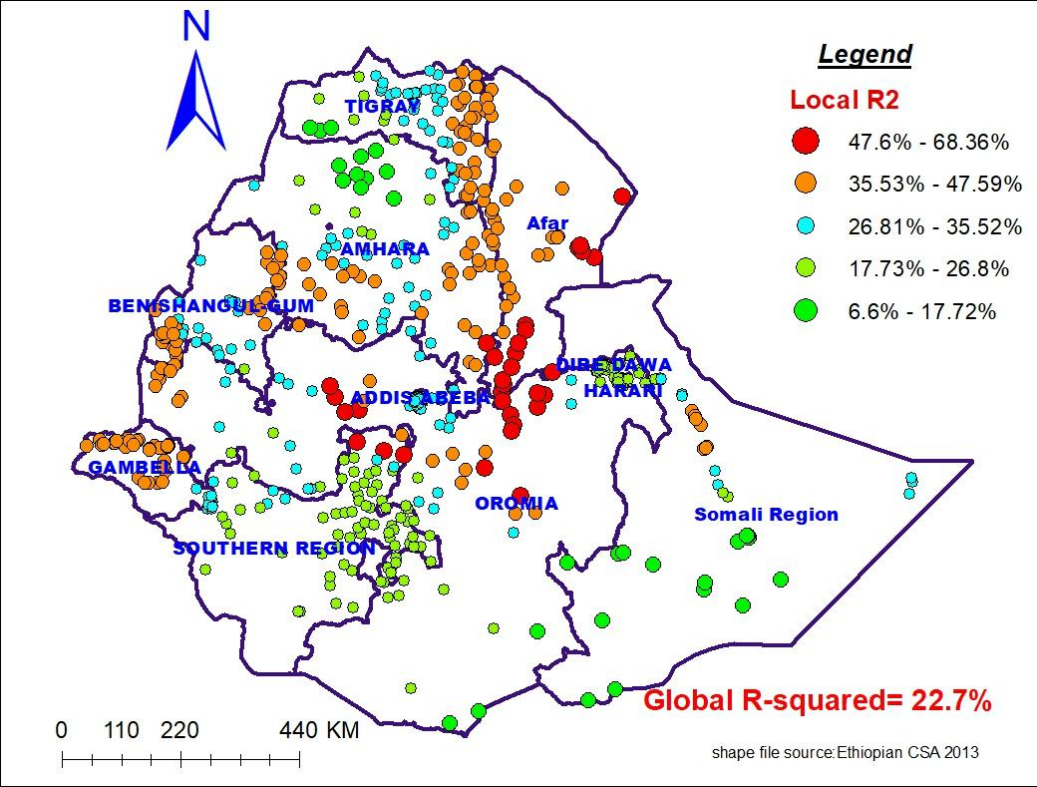
The spatial mapping of local adjusted R-square of GWR model in Ethiopia, 2016

Figure 6 denotes that the model performance (local R-squared) mapped over the study area. Good performance of the model i.e. R-squared ranging from 41 to 68.3% was shown northwest of Gambela, western and central Oromia, all portion of Afar, southeast Amhara and eastern Tigray regions. The model has a poor fit with data for provinces located in most parts of SNNP, southern part of Somali, northern and central part of Amhara region (i.e. adjusted R^2^ ranging 6.6–27%) (Fig. 6).

**Fig. 7:**
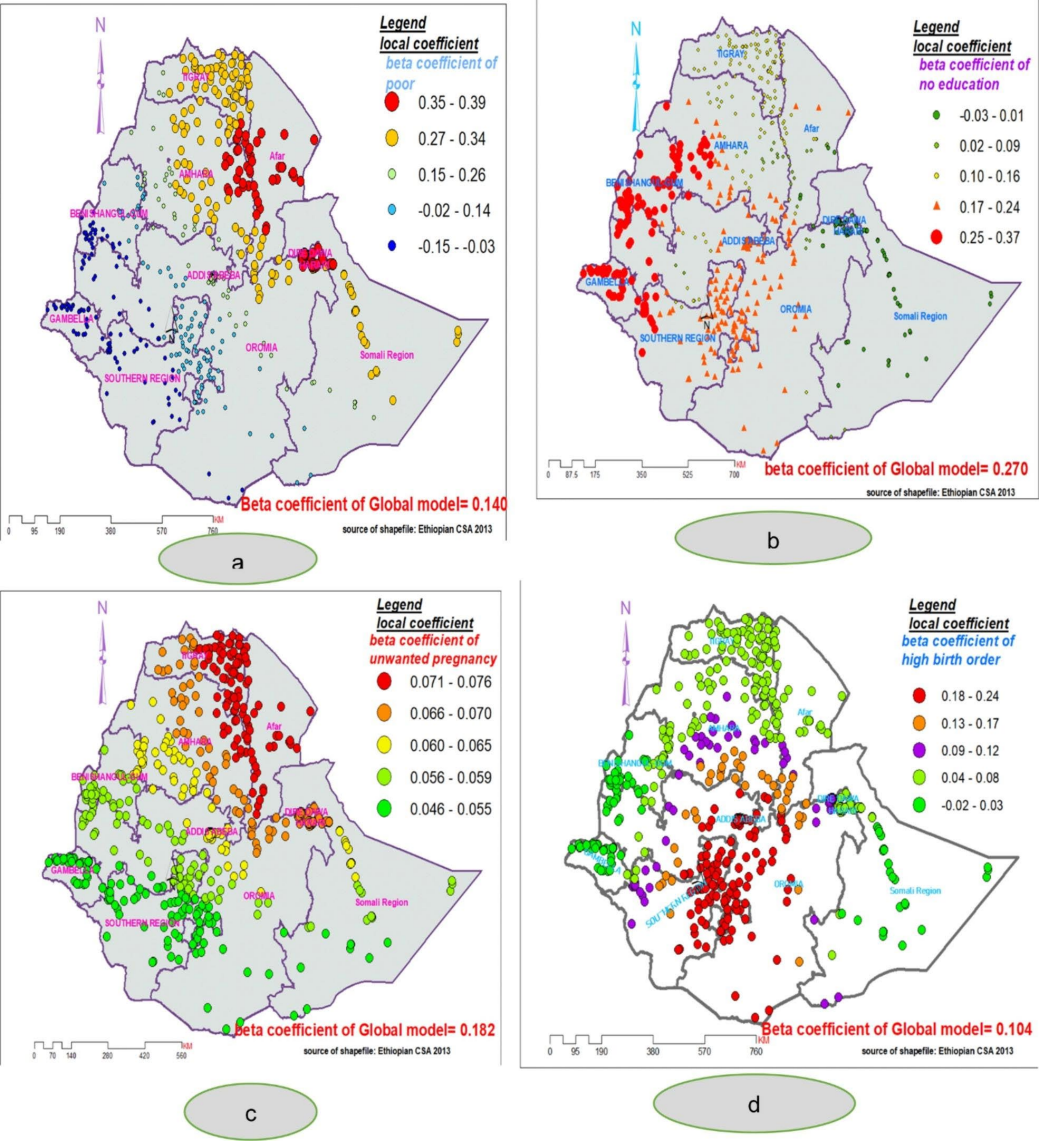
The spatial mapping of local regression coefficients of poor wealth (**a**), no education (**b**), unplanned last pregnancy (**c**) and higher birth order (**d**) in Ethiopia, 2016

Figure 7 (A, B, C & D) shows the results of the spatial distribution of the beta coefficients of the four explanatory variables. The red dotted area represent a strong influence (high beta coefficient) of the explanatory variables on delayed ANC initiation. Beta coefficient of being not educated women shows variation across the study area, indicating that an inconsistent relationship between not being educated and the proportion of delayed ANC initiation across the study area. A strong positive influence of being not educated women on delayed ANC was shown in whole parts of Gambela, Benishangul, Gumuz and SNNP regions. It also covers some parts of Oromia (western, south, and southwest), southern and southwest of Amhara Region, and some parts of Afar Region. The lowest coefficient for women with no education was noticed in North-East part of Ethiopia (north-East Amhara, entire part of Tigray, East Oromia, and whole part of Somalia region) (Fig. 7b).

Regarding the beta coefficient of women from poor household wealth status, a strong and positive influence was found concentrated in most parts of Afar, Somali, and Amhara regions. The value of beta coefficient ranges from 0.27 to 0.39 units in those areas (Fig. 7a). In the same way, stronger influence of unplanned pregnancy on delayed ANC initiation was noticed in the whole parts of (Amhara, Tigray, and Afar), some parts of southern Oromia and north of Somali region (Fig. 7c).

Besides, having a higher birth order (birth of four and above) was associated with the spatial variation of delayed ANC initiation. A strong and positive relationship between having a birth order and delayed ANC initiation was identified in south Amhara, most parts of Oromia( except Western portion), and most parts of SNNP Somalia region. Vice versa, weak relationship was observed in all parts of Tigray, Somali, Benishangul, Gambela, and Amhara region (Fig. 7d).

## Discussion

This study aims to determine the spatial pattern and spatial non-stationary of delayed ANC initiation in Ethiopia. The findings of the spatial global Morn’s analysis showed that the proportion of delayed ANC initiation showed spatial variation (clustering) in Ethiopia. This finding is in agreement with a study conducted in Nigeria [44], which found that being a northern resident was related to a high delayed ANC rate. Another supportive finding from Nepal [47] concluded that living in the far western region of the country was more likely to initiate ANC than women residing in the Eastern region.

The significant hotspot area of delayed ANC initiation (primary cluster) was detected in the administrative zones of SNNP and Oromia regions. Prior pocket studies also announced a high rate of delayed ANC initiation in that area [48–51]. This clustering might be also derived from the insufficiency of resources and the asymmetrical distribution of health facilities and other components of the health system. The evidence disclosed that health care access challenges were found to be clustered in those listed hotspot areas, mainly in the areas covered by the most likely cluster and the secondary cluster 1(Fig. 5) [51].

In the local model, no formal education, poor household wealth status, unplanned last pregnancy, and higher birth order were the significant explanatory variables of the observed spatial variation of delayed ANC initiation in Ethiopia. The variables having higher birth order and being uneducated women in this spatial regression model were also found to be explanatory variables of delayed ANC initiation using multinomial logistic regression analysis conducted in this study.

The strong and positive relationship of no education with the hotspot areas in Oromia, Benishangul, Gambela, and SNNP might be due to uneducated women being more likely to delay their first ANC visit [18]. Additionally, less educated women are less likely to have awareness about the importance of initiating antenatal care as early as recommended per the WHO schedule and are less likely to take part in decision-making activities concerning their health [52]. The results of this study have a variety of practical implications for government and policymakers, including the need to engage women in decision-making and assigning additional resources to raise maternal health awareness. Moreover, compared to women who had planned the last pregnancy, women who had unplanned last pregnancy had a positive relationship with the hotspot of delayed ANC in the central part of Tigray. This finding were approved by other studies in Ethiopia [20, 53]. The strong relationship between unplanned pregnancy and delayed ANC distinguished in the Afar region might be related to low family planning utilization [54] and decision-making autonomy regarding family planning [52, 55].

Positive and solid association between poor wealth index and delayed ANC initiation in most parts of southeast Amhara, entire parts of Afar, and Eastern Somalia regions. These relationships may partly be explained by; low utilization of maternal health services in those areas [54]. And poor maternal service uptake is more likely to be correlated with poor wealth status because of the cost of transport to reach health facilities. The current study also identified a strong relationship between having a higher birth order and hotspots of delayed ANC initiation in limited parts of Oromia and most parts of SNNP regions. A previous study also reported that as the birth order gets higher, the rate of maternal health service uptake decreases accordingly [56]. A possible explanation may be that women with higher birth order are too late in attending ANC timely because of engagement in nursing the extended family member.

This study utilized nationwide representative data that gives better generalizability. The hierarchical nature of sampling was accounted for by applying STATA “svyset” command for each descriptive and analytical analysis. The spatial heterogeneity of the model parameters has been accounted for. Moreover, the statistically significant hotspot area detected by using Kulldorff’s spatial scan statistical tests and hotspot analysis using ArcGIS software converging to each other were the main strengths of this study and were the main strengths of this study.

However, the results of this study shall be comprehended based on the context of succeeding limitations. As we use secondary data, important explanatory variables like cost of transport services and means of pregnancy recognition, provider’s gender, and preference of mother, health services, quality, and satisfaction level are not incorporated. Additionally, this study used data from a cross-sectional study design, which is difficult to declare causality between the outcome variable and the explanatory variables. This survey interviewed women who gave birth five years before the interview time, recall bias is inevitable. The relationship between delayed ANC initiation and health insurance enrollment is unclear and the justification that we gave may not satisfactory and might need additional exploration.

## Conclusion

In this study, the spatial pattern of delayed antenatal care initiation in Ethiopia is clustered (i.e. Moran’s I value = 0.385, p-value < 0.001). Statistical significant clustering of delayed antenatal care was situated in the administrative zones of Oromia, SNNPR, Benshangul-gumuz, and Gambela and Tigray regions. Therefore, location-based interventional strategies are required to halt delayed ANC initiation in a more cost-effective way rather than simply providing the service randomly.

The geographic weighted regression model (MGWR) shows that the explanatory variables (being uneducated women, respondents with poor household wealth status, those having unplanned last pregnancy, and higher birth order) had significant influences on the spatial variation of delayed ANC.

## Declarations

**Availability and data and materials**.

The data which the authors used to produce this manuscript, the data set can be accessed from the Demographic and Health Surveys (DHS) Program by requesting for this work and accessing www.dhsprogram.comwebsite and the datasets used and/or analyzed during the current study are available from the corresponding author on reasonable request.

## Abbreviations

**ANC**: Antenatal Care
**DHS**: Demographic and Health Survey
**EAs**: Enumeration Areas
**EDHS**: Ethiopian Demographic and Health Survey
**GIS**: Geographic Information System
**GWR**: Geographically Weighted Regression
**LLR**: Log-Likelihood Ratio
**ME**/ **MPE**: Mean Error/ Mean Predicted Error
**MGWR**: Multiscale Geographic Weighted Regression
**OK**: Ordinary Kriging
**OLS**: Ordinary Least Square
**RMSE**/**RMSPE**: Root Mean Squared Error/ Root Mean Squared Predicted Error
**RRR**: Relative Risk Ratio
**SNNP**: South Nation and Nationalities People
**VIF**: Variance Inflation Factor
